# Vacilación ante la vacuna contra el covid-19 en Estados Unidos de América: un estudio etnográfico digital

**DOI:** 10.18294/sc.2024.4541

**Published:** 2024-03-26

**Authors:** Rosalynn Adeline Vega

**Affiliations:** 1 Doctora en Antropología Médica. Profesora asociada, University of Texas Rio Grande Valle, Edinburg, EEUU. Rosalynn.Vega@utrgv.edu University of Texas Rio Grande Valle University of Texas Rio Grande Valle Edinburg USA Rosalynn.Vega@utrgv.edu

**Keywords:** Vacuna COVID-19, Niño, Padres, Pediatras, Estados Unidos de América

## Abstract

Luego de que se autorizara en EEUU el uso de la vacuna contra el covid-19 en bebés de seis meses a niños y niñas de cuatro años, algunas personas (padres, madres, pediatras y comunicadores) plantearon la vacunación contra el covid-19 como una cuestión de acceso; sin embargo, muchas otras se mostraron reacias y otras se resistieron a las recomendaciones de los Centers for Disease Control and Prevention de EEUU. En este contexto, este estudio se propuso explorar: 1) reacciones divergentes ante la autorización de uso de la vacuna contra el covid-19 en niños y niñas de seis meses a cuatro años; y 2) lógicas contrapuestas que subyacen a las actitudes provacunación, antivacunación y vacilación ante las vacunas contra el covid-19. Para ello, se realizó una etnografía digital, con monitoreo de 5.700 reacciones a una serie de ocho infografías publicadas en las redes sociales por la John Hopkins Bloomberg School of Public Health, y observación participante en un grupo focal en línea a lo largo de un año, desde diciembre de 2021 hasta diciembre de 2022, conformado por 18 madres. Los resultados indican que el personal médico debe considerar diferentes nociones de “riesgo” al interactuar con los pacientes, especialmente aquellos que dudan en vacunarse.

## PRESENTACIÓN DEL TEMA

El 17 de junio de 2022, la Food & Drug Administration (FDA) de EEUU autorizó el uso de las vacunas contra el covid-19 de Moderna y Pfizer-BioNTech en niños a partir de los seis meses de edad^1^. Al día siguiente, el 18 de junio, la directora de los Centers for Disease Control and Prevention (CDC), Rochelle P. Walensky, respaldó la recomendación del Advisory Committee on Immunization Practices (ACIP) de que todos los niños de seis meses a cuatro años de edad recibieran la vacuna contra el covid-19. En el período previo a la autorización de la FDA, algunos padres, pediatras y medios de comunicación plantearon la vacunación contra la covid-19 como una cuestión de acceso^3^. Sin embargo, los estudios no lograron demostrar que vacunar a bebés y niños muy pequeños reduzca significativamente los casos graves de infección (es decir, los casos que resultan en hospitalización y muerte). 

El documento informativo de la FDA del 15 de junio de 2022, dice: “En los grupos de mayor edad pediátrica, se ha demostrado que la vacuna previene la hospitalización y otras secuelas graves [...] la implementación de la vacuna para su uso en niños de 6 meses a 4 años de edad probablemente tendrá un efecto beneficioso en la morbilidad y mortalidad asociadas con covid-19 en este grupo de edad”^4^. Este documento de la FDA infiere un beneficio probable para los niños pequeños, basándose en el beneficio demostrado para los grupos de edad pediátrica mayor; sin embargo, no presenta ningún dato que demuestre que la vacunación sea beneficiosa para niños de 6 meses a 4 años. 

Dos meses después de la aprobación de la FDA, el artículo de Anne Hause *et al*. se centró en la seguridad posterior a la autorización de la serie primaria de la vacuna contra el covid-19 en niños pequeños, a través de la revisión de eventos adversos e impactos en la salud, después de la vacunación covid-19^5^. De 5.011 niños de 6 meses a 5 años incluidos en el estudio, se informaron 1.017 eventos adversos. Si bien este artículo indica que el 98,1% de los eventos se clasificaron como no graves y el 1,9% como graves (lo que significa que se esperan reacciones después de la vacunación, pero los eventos adversos graves son raros), el estudio no investiga si la vacunación tiene un efecto beneficioso sobre la morbilidad y la mortalidad. Si no se ha demostrado que la vacunación contra el covid-19 sea beneficiosa para los niños pequeños, En lugar de “proceso de pensamiento”, podrías usar “razonamiento” o “forma de pensar”. Por lo tanto, la frase podría ser: ¿cuáles son las preocupaciones, valores y razonamientos de los padres a favor de la vacunación?

Por otro lado, las dudas sobre las vacunas son cada vez más frecuentes en EEUU. Después de la aprobación de la vacuna contra el covid-19 para niños pequeños^1^ el 18 de junio de 2022, muchos padres y madres se mostraron reacios y algunos se resistieron a la recomendación de los CDC. De hecho, el 21 de noviembre de 2022, según la infografía publicada por la John Hopkins Bloomberg School of Public Health^6^^)^ solo el 9% de niños y niñas elegibles habían recibido al menos una dosis de la vacuna. En otra infografía publicada el mismo día por la misma institución, se lee: “Why aren’t kids under 5 getting vaccinated for Covid?”^7^ (¿Por qué no se están vacunando contra el covid los niños menores de 5 años?). La pregunta que surge es ¿qué factores hacen que los padres duden a la hora de vacunar a sus hijos?

### Provacunación, antivacunas y vacilación ante las vacunas

El término *provacunación* describe a la “mayoría silenciosa” que considera la vacunación como un bien público indiscutible^8^. Kashyap *et al*.^9^ caracterizan a este grupo como aquellos que aceptan fácilmente la vacunación debido a una fe implícita en el gobierno, su sistema de salud y sus escuelas públicas. Las vacunas están ampliamente asociadas con el progreso tecnológico, la medicina moderna y una atención de salud pública rentable.

Según Kashyap *et al*.^9^, el movimiento antivacunas está constituido por una pequeña minoría, pero ruidosa, de los llamados “desafiantes” que comparten una visión del mundo y una ideología médica que ignora la vacunación como antídoto. Vogel^10^ describe a este grupo como el 2% que rechaza rotundamente las vacunas y no se deja convencer de lo contrario. El movimiento antivacunas se ha caracterizado como una guerra entre pediatras y pacientes^11^. Padres y madres antivacunas a menudo suscriben filosofías científicas alternativas o tienen creencias conspirativas que reflejan una desconfianza profundamente arraigada en el gobierno (por ejemplo, las vacunas están diseñadas para reducir las poblaciones minoritarias, hacer que niños y niñas sean impotentes o infértiles y beneficiar los intereses comerciales de las compañías farmacéuticas). En el contexto de la pandemia de covid-19, los grupos antivacunas organizados sostenían que el covid-19 no era peligroso, pero que la vacuna sí lo era, y que no se podía confiar en los defensores de las vacunas^12^.

En cuanto a la *vacilación ante las vacunas*, un número cada vez mayor de personas cuestiona la necesidad y la seguridad de las vacunas. Charles^13^ señala que la sospecha sobre las vacunas es distinto al rechazo, y que también se aleja de las creencias antivacunas. Larson y Broniatowski^12^ indican que las personas que dudan en vacunarse son aquellas que están indecisas. Aunque la indecisión ante posibles riesgos para la seguridad de las vacunas no es lo mismo que estar en contra de las vacunas, las personas que dudan en vacunarse corren el riesgo de ser estigmatizadas como “antivacunas” por los profesionales de la salud.

En realidad, Rozbroj *et al*.^14^ señalan que muchos padres y madres que dudan en vacunar a sus hijas e hijos se preocupan por las vacunas ante el calendario recomendado por los CDC (por ejemplo, la administración de la vacuna contra la hepatitis B en bebés, a pesar de que la hepatitis B se transmite por sangre, semen u otros fluidos corporales a través del contacto sexual, el uso de drogas inyectables, etc.). Estas personas pueden simplemente retrasar la recepción de una vacuna debido a la ansiedad que les genera las cuestiones de seguridad, para finalmente recibirla. Aunque la mayoría de las madres que dudan, deciden en última instancia seguir el calendario de vacunación recomendado para sus hijos e hijas, siguen sintiéndose ambivalentes acerca de su decisión^15^.

Las tasas de vacunación están disminuyendo y los brotes de enfermedades prevenibles han aumentado en algunas áreas de EEUU. En 2015, un brote de sarampión que se originó en Disneyland, California, se extendió a otros seis estados de EEUU, a México y Canadá^16^. Ese mismo año, California aprobó el Proyecto de Ley del Senado 277, que eliminó la opción de exención por creencias personales para las vacunas al ingresar a la escuela. Al hacerlo, California se convirtió en el tercer estado de EEUU en eliminar las exenciones no médicas.

Más recientemente, el aumento de las dudas sobre las vacunas contra el covid-19 han provocado disminuciones en la cobertura de vacunación en todo EEUU^17^. Williams y O’Leary estiman que el umbral de cobertura de vacunación necesario para prevenir brotes, como el sarampión, es del 95%. Durante el año escolar 2021-2022, la cobertura de las vacunas contra la rubéola, las dos dosis de sarampión y paperas entre niños y niñas de jardín de infantes cayó al 93%, la más baja en una década^17^. El Distrito de Columbia tenía menos del 90% de cobertura. A escala global, el aumento del rechazo a las vacunas entre los países desarrollados y en desarrollo ha llevado a la Organización Mundial de la Salud a declarar la disminución de la vacunación como una de las diez principales amenazas para la salud mundial^18^.

Si bien muchos padres y madres aceptaron fácilmente la vacuna contra el covid-19 para sus hijos pequeños, una minoría considerable se negó o retrasó la aceptación de la vacuna. Ante este escenario, este artículo se propone abordar las siguientes dimensiones: 1) reacciones divergentes ante la autorización de uso de la vacuna contra el covid-19 en niños y niñas de seis meses a cuatro años; y 2) lógicas contrapuestas que subyacen a las actitudes provacunación, antivacunación y vacilación ante las vacunas contra el covid-19.

## METODOLOGÍA

Para explorar las actitudes de padres y madres con respecto a las vacunas pediátricas contra el covid-19, realizamos una etnografía virtual, que Hine define como etnografía realizada “en, de y a través de lo virtual”^19^. Las etnografías estudian cada vez más las prácticas, temas, grupos y modos de comunicación que dependen totalmente de las tecnologías digitales para su existencia^20^^,^^21^^,^^22^^,^^23^^,^^24^^,^^25^^,^^26^^,^^27^. Aunque la etnografía virtual consiste en un “desplazamiento experiencial más que físico”^28^, el principio etnográfico clave es desarrollar una comprensión de los fenómenos sociales en cuestión a través de la observación participante (es decir, la inmersión) y la recopilación progresiva de datos (por ejemplo, la investigación sistematizada) sigue siendo consistente con la etnografía tradicional^19^. Además, si bien la descorporeización de las interacciones digitales puede hacer que el mundo en línea parezca un “no espacio”^29^, la investigación etnográfica demuestra cómo las colectividades sociales habitan en los medios digitales como una “ubicación cultural” a través de nuevos modos de comunicación^30^^,^^31^. Por lo tanto, la etnografía puede aprovecharse como una herramienta valiosa para analizar comunidades sociales complejas en línea.

En los días posteriores a que la FDA autorizara el uso de las vacunas contra el covid-19 de Moderna y Pfizer-BioNTech en niños y niñas, por un lado, monitoreamos un total de 5.700 reacciones a una serie de ocho infografías publicadas en las redes sociales por la Johns Hopkins Bloomberg School of Public Health sobre la autorización del 18 de junio de 2022 de la vacuna contra el covid-19 para niños y niñas de seis meses a cuatro años. Si bien el grupo focal en línea que creamos permitió un debate íntimo de persona a persona, el análisis de las reacciones en las redes sociales a las infografías captura las actitudes de una muestra más grande, lo que Airoldi^32^ podría llamar un “metacampo”. Además, el análisis de las redes sociales ofrece información valiosa, ya que las prácticas de las redes sociales a menudo reflejan cómo los participantes de la investigación etnográfica navegan por el mundo social y material más amplio^33^. Por otro, realizamos observación participante en un foro en línea, a través de la aplicación Peanut, diseñada para madres que criaban a sus hijos e hijas durante la pandemia de covid-19. Si bien la aplicación Peanut está disponible para usuarias de Android e iOS de todo el mundo, la gran mayoría de las mujeres residen en EEUU. Creamos un hilo preguntando si los participantes estaban planeando vacunar a sus hijos contra el covid-19 y por qué, creando así un grupo focal en línea. Al hacerlo, creamos un objeto focal (es decir, las actitudes de padres y madres hacia la vacunación contra el covid-19 para niños y niñas de seis meses a cuatro años) dentro de un campo “contextual” relativamente estable (es decir, el foro en línea de la aplicación Peanut, que Airoldi^32^ y Caliandro^34^ se refieren a ella como una comunidad en línea “clásica”. En este hilo, fueron 18 personas las que discutieron qué los motiva a buscar la vacuna contra el covid-19 para sus hijos e hijas pequeños o, alternativamente, por qué se resistían a la reciente recomendación de los CDC. Las personas participantes no solo ofrecieron su respuesta personal a la pregunta, sino que muchas participaron entre sí en un debate continuo sobre el tema. Las 45 publicaciones del hilo se incluyeron como puntos de datos para este estudio.

Optamos por una participación “activa” en este grupo (es decir, crear un hilo dentro del grupo, constituyendo así un grupo focal en línea) en lugar de un “acecho” discreto en línea. Hine^28^ señala que la etnografía virtual discreta (es decir, observar interacciones en línea sin intentar interactuar con los miembros) es considerablemente atractiva para algunos etnógrafos ya que proporciona un medio para estudiar la vida social tal como se vive; sin embargo, también presentan problemas éticos^35^. Steinmetz^36^ llega incluso a argumentar que cuando un investigador “acecha” en un foro en línea sin participar, no está realizando datos etnográficos primarios, sino más bien un análisis de contenido secundario. En lugar de investigación etnográfica, “al acecho” se describe mejor como investigación de archivos. Por el contrario, la participación activa en un grupo ayuda a los etnógrafos virtuales a adquirir conocimiento experiencial sobre las interacciones grupales, al mismo tiempo que abre otras formas de interacción en línea entre los participantes del grupo y el etnógrafo, que se perderían si quien realiza la etnografía se limitara a la observación discreta de las transmisiones públicas.

Nuestra decisión de buscar una participación “activa” refleja nuestro compromiso con la ética en la etnografía en línea y nuestros esfuerzos por desarrollar la confianza con los participantes. Según Hine^28^, la confianza se desarrolla cuando la presencia del etnógrafo es aceptable para los miembros de la comunidad social en línea. Al mismo tiempo, los etnógrafos virtuales pueden determinar si lo que están observando es auténtico (en contraposición a una mera exhibición para su beneficio) a través de una presencia continua en el grupo en línea. Aunque los datos incluidos en este artículo se limitan al grupo focal que creamos, nuestra observación participante en el foro en línea duró un año. Esta presencia continua nos permitió desarrollar la confianza con las personas participantes y determinar la autenticidad de las interacciones que observamos. Nuestra participación “activa” se hizo explícita ante el grupo, junto con las características de la investigación. Se informó a los participantes que sus interacciones se tomarían como material de investigación y que se salvaguardaría el anonimato en todo momento.

Los etnógrafos virtuales deben entender cómo analizar la vida social y material de los medios digitales, dada la naturaleza efímera, mutable, hipermóvil y anónima de los datos digitales, como los memes de Internet, los chats, las publicaciones en las redes sociales y los comentarios que los acompañan^37^^,^^38^. Además de la observación participante y de las entrevistas realizadas en línea, la etnografía virtual también puede incluir técnicas complementarias como la recopilación de capturas de pantalla, la captura de registros de chat y el análisis de publicaciones en redes sociales^39^. En este estudio, recopilamos capturas de pantalla del debate que se desarrolló en el hilo que creamos sobre las actitudes de los padres hacia la vacunación pediátrica contra el covid-19. 

Sin embargo, la etnografía virtual sobre temas relacionados con la salud plantea cuestiones éticas sobre la privacidad de la persona usuaria/paciente y la salud pública, particularmente en torno al uso de datos de identificación individual que pueden ser (semi)privados. Un representante del Institutional Review Board for Human Subjects Research de la University of Texas Rio Grande Valley determinó que, dada la naturaleza del foro en línea, no era necesario enviar el protocolo de investigación para su revisión. El foro en línea constituye un espacio público en línea al que pueden acceder fácilmente personas de todo el mundo sin costo alguno. Además, las personas involucradas eran anónimas, incluso para nosotros. Las personas se identificaban a través de un simple identificador específico del foro (que puede ser un nombre o un alias). Nadie en el foro podía acceder a información de identificación como apellidos e información de contacto (por ejemplo, números de teléfono, direcciones de correo electrónico y domicilios particulares). La aplicación no contiene una base de datos de usuarios con capacidad de búsqueda. A pesar de este anonimato inherente, somos sensibles a las expectativas de los informantes en línea, incluso cuando sus actividades ocurren en espacios públicos en línea^40^. Para preservar aún más el anonimato de las personas involucradas en el debate, omitimos las citas directas de los comentarios de las personas, las capturas de pantalla y el nombre de usuario que utilizaron en el foro. Todos los nombres utilizados en este artículo son seudónimos.

Después de observar cómo se desarrollaba el debate dentro del grupo focal y observar las reacciones en las redes sociales a las infografías publicadas por la Escuela de Salud Pública Bloomberg de Johns Hopkins, utilizamos un proceso iterativo de codificación abierta para identificar temas emergentes. Continué con una revisión de la literatura sobre las dudas sobre las vacunas para comprender mejor cómo la recomendación de la vacunación contra el covid-19 para niños pequeños representa un elemento “inquietante” en un panorama de vacunación pediátrica ya tumultuoso.

## RESULTADOS

### Reacciones divergentes ante la autorización de uso de la vacuna contra el covid-19 en niños y niñas de seis meses a cuatro años

Observamos reacciones encontradas a una serie de ocho infografías publicadas en Facebook por la Johns Hopkins Bloomberg School of Public Health sobre la autorización del 18 de junio de 2022 de la vacuna contra el covid-19 para niños de seis meses a cuatro años. Una de las infografías en línea advierte a padres y madres que, si se infectan con covid-19, sus hijas e hijos pueden experimentar síntomas graves y consecuencias para su salud a corto y largo plazo. La infografía continúa indicando que, aunque los casos graves de covid-19 entre niños y niñas son raros, casi 1.500 han muerto debido a la infección por covid-19 en los EEUU. En esta infografía, se pone énfasis en las consecuencias para la salud a corto y largo plazo y en las 1.500 muertes infantiles (estas partes aparecen en negrita). Esta infografía se combina con otras infografías que descartan los temores de que la vacuna contra el covid-19 sea potencialmente insegura o cause efectos secundarios, calificándolos de “escepticismo injustificado” e “ideas falsas”.

Si bien la mayoría de las publicaciones de la Johns Hopkins Bloomberg School of Public Health genera menos de cien reacciones, la publicación que contiene infografías sobre la vacunación contra el covid-19 para niños pequeños generó 5.700 reacciones, 1.200 comentarios y 664 acciones. Del total de reacciones, al 34,5% le “gustó” la publicación, al 34,5% le “enojó”, al 16,1% le “encantó”, el 10,4% “se rio”, al 2,2% le “entristeció”, >1% colocó el emoji que simboliza “apoyo y cuidado” (abraza un corazón), y >1% estaban “conmocionados”. Asimismo, los comentarios en la publicación variaron desde la aprobación hasta el enojo y la burla. Destacamos a las personas cuyos comentarios generaron un acalorado debate y nos adentramos en la naturaleza de esos debates.

#### Sandra

El comentario que generó más reacciones (2,9k reacciones, incluyendo “pulgar arriba”, corazón y risas) fue de Sandra, madre de dos niños pequeños. Sandra reveló que su familia no está vacunada y ha tenido covid-19 tres veces. Además, indicó que ella y sus hijos se han recuperado cada vez más rápido que su madre, a pesar de que su madre fue vacunada contra el covid-19. Las respuestas a este comentario fueron mixtas. Algunos apoyaron a Sandra, ofreciendo ejemplos de cómo la vacuna no protege a las personas de contraer o propagar la enfermedad, e indicando que sus experiencias personales con el covid-19 fueron menos graves que la gripe estacional o “una resaca”. Otros juzgaron a Sandra por transmitir la enfermedad a otros miembros de la comunidad, incluida su anciana madre, que corre un mayor riesgo de sufrir complicaciones de salud graves o incluso la muerte.

Sandra y su familia ofrecen un ejemplo de cómo la toma de decisiones sobre vacunación es un proceso relacional. Las decisiones sobre vacunación se ven afectadas por las relaciones con los demás, de la misma manera que las relaciones con los demás se ven afectadas por las decisiones de vacunación.

Quienes apoyaron a Sandra crearon cohesión dentro del grupo a través de su rechazo compartido a la vacuna contra covid-19. En esta línea, Elisa Sobo^41^ destaca cuán productivo es el rechazo a las vacunas para las relaciones sociales inmediatas. Esta autora sostiene que el rechazo no solo niega las definiciones exogrupales, sino que también reafirma los marcos intragrupales y, por lo tanto, sus vínculos. En la misma línea, Charles^13^ indica que la sospecha vacunal es generadora de relaciones y afectos.

Quienes criticaron a Sandra por su decisión de no vacunarse, culpándola de propagar el covid-19 a otros miembros de la comunidad y a la madre de Sandra, se hicieron eco de un tema subyacente en una de las infografías analizadas que representa un tornado de gérmenes acercándose a una casa. La infografía explica que la infección por covid-19 es perjudicial para el hogar ya que puede propagarse entre la familia y que vacunar a niños y niñas pequeños puede brindar “tranquilidad” a la familia. Este marco apunta a los niños y las niñas como ciudadanos biológicos que pueden desempeñar un papel en la protección a una mayor propagación del covid-19 en la población adulta. Es decir, este cambio replantea la concepción de niños y niñas que pasan a ser ya no solo receptores de cuidados, sino ciudadanos encargados del cuidado de los demás.

#### Anya

El comentario que generó la segunda mayor cantidad de reacciones (1,7K de reacciones, incluidos “pulgar hacia arriba”, risa y llanto) fue el de Anya, una madre que recibió su vacuna de refuerzo contra el covid-19 durante el embarazo y cree que es importante mantener a sus hijos completamente inmunizados. Toda su familia de cinco miembros nunca ha dado positivo por covid-19 u otros virus, y sus hijos seguirán recibiendo todas las vacunas recomendadas. Mientras tanto, otra madre llamada Elise respondió a Anya ofreciendo evidencia contradictoria a la experiencia de Anya: nadie en su familia de seis miembros ha sido vacunado y nadie ha contraído covid-19.

Otros usuarios dejaron comentarios irónicos acerca de enviarle los mejores deseos a Anya por las enfermedades que desarrollará su familia al haber recibido las vacunas. Una mujer llamada Rosa le respondió diciéndole que Anya se arrepentirá de su decisión en el futuro cuando descubra que sus hijos son estériles. Otra usuaria comentó que está rezando para que sus hijos sigan vivos dentro de 20 o 30 años. Estos comentarios recibieron “aprobado”, “amor” y reacciones de risa. Otros sugirieron que al aceptar la vacuna contra el covid-19 para sus hijos e hijas pequeños, Anya estaría donando a sus hijos a la ciencia e inscribiéndolos tempranamente en un programa de eugenesia.

Otros respondieron que Anya incluiría a sus hijos en el grupo de “intervención” de un ensayo clínico aplicado a toda la sociedad para una vacuna que ha sido aprobada sin pruebas suficientes. En el caso de niños pequeños de Anya, un usuario indicó que, al aceptar la vacuna, Anya estaría tomando a un niño que tiene un riesgo mínimo de complicaciones graves por covid-19 y colocándolo en un estudio a largo plazo con consecuencias de salud desconocidas para su futuro. 

Sorell y Butler^42^ sostienen que las redes sociales han facilitado la difusión de teorías de conspiración en torno al covid-19 y los programas de vacunación masiva. Sin embargo, May Goldberg sugiere en su libro *Vaccine hesitancy: public trust, expertise, and the war on science*^43^ que en nuestra era de política científica, el lenguaje de la ciencia es la moneda del discurso político. Por tanto, los debates sobre qué conocimiento científico debería producirse y cómo utilizarlo sustituyen los debates sobre valores. Las perspectivas sobre la vacunación tienen una carga social, ya que abordan cuestiones sexuales, de mujeres, religiosas, de políticas relacionadas con la salud a nivel provincial y federal, y con las grandes farmacéuticas^11^.

Goldberg sostiene que las dudas sobre las vacunas se han planteado incorrectamente como resultado de una “guerra contra la ciencia”. En este marco contradictorio de la vacilación ante las vacunas, individuos ignorantes, científicamente analfabetos e irracionales se oponen a los expertos científicos, o sus sesgos cognitivos sustentan la vacilación ante las vacunas frente a la evidencia. Sin embargo, Goldberg sostiene que lo que impulsa la duda sobre las vacunas no es un aumento del sentimiento antiexperto o un “populismo epistemológico” en el que cada uno es su propio experto y “hace su propia investigación”, sino más bien una falta de confianza en las instituciones científicas. Es decir, la desconfianza en las instituciones científicas puede impulsar a los padres a cuestionar la autoridad biomédica y desmedicalizar sus enfoques de la salud^16^.

Sin transparencia por parte de las instituciones científicas y sin confianza del público, la ciencia no puede guiar eficazmente las políticas. En este contexto, fortalecer la credibilidad de las vacunas mediante una mayor confianza en las instituciones científicas sería más eficaz que las medidas existentes para fomentar la cobertura vacunal^44^. Hasta ahora, la institución médica ha intentado generar confianza centrándose en la comunicación entre pacientes y proveedores en lugar de abordar la confianza pública general en las vacunas infantiles. Lauren Vogel^10^ aconseja que los proveedores concentren sus esfuerzos en generar confianza con los llamados “cuidadores de la valla” de las vacunas^10^. En lugar de centrar sus esfuerzos en los padres y las madres que rechazan rotundamente las vacunas, Vogel sugiere que los proveedores se centren en convencer a quienes dudan sobre las vacunas para que acepten recomendarlas brindándoles información, ofreciéndoles garantías y utilizando sus propias historias de encuentros con enfermedades prevenibles con vacunas. Este enfoque se guía por estimaciones de que menos del 2% de padres y madres son “antivacunas”, mientras que hasta un 30% se muestran reacios a vacunarse^45^. Desafortunadamente, las estrategias de Vogel para convencer a las personas que dudan de las recomendaciones de los CDC no muestran un acercamiento y una comprensión del otro, ya que su enfoque para “generar confianza” no propone que los proveedores consideren cuidadosamente las razones que sustentan las dudas de padres y madres y comprendan que las dudas sobre las vacunas no se deben simplemente a una falta de información.

Cuando la desconfianza en el gobierno contribuye a las dudas sobre las vacunas, esta desconfianza puede atribuirse a experiencias comunitarias históricas, especialmente entre las comunidades BIPOC (black, indigenous, people of color)^12^^,^^46^^,^^47^. Es posible que el público no confíe en la comunidad científica debido a una historia de racismo médico o a la comercialización de las ciencias (que priorizan las ganancias sobre la seguridad), especialmente, la ciencia médica. Las injusticias específicas incluyen la eugenesia, las cuarentenas de grupos minoritarios, la destrucción de barrios “insalubres” ocupados por comunidades marginalizadas, la crisis de opioides e investigaciones poco éticas como el experimento de Tuskegee^46^. Kasstan^48^ encuadra las dudas sobre las vacunas como parte de un cambio más amplio en las relaciones de la salud pública con los grupos minoritarios. Sostiene que una mejor comprensión de la toma de decisiones sobre vacunas ayudará a evitar representaciones dañinas de las minorías y, al mismo tiempo, abordará las dudas sobre las vacunas de manera más sostenible y confiable.

#### Tilda

Una madre, a la que llamaremos Tilda, contó cómo la vacuna contra el covid-19 había sido alentada, luego incentivada y finalmente impuesta a la población adulta de EEUU. Argumentó que, como resultado de esto, los padres no vacunarán voluntariamente a sus hijos contra el covid-19. Majid y Ahmad^49^ señalan cómo las presiones extremas para vacunar en forma de sanciones financieras (es decir, no recibir estipendios gubernamentales para el cuidado infantil) y exclusión (es decir, no ser aceptado en la escuela pública) llevaron a oportunidades limitadas de aprendizaje para los niños de padres que dudan en vacunarse. En este sentido, Kashyap *et al*.^9^ indican que sentirse forzado por las autoridades escolares, las malas relaciones entre padres, madres y proveedores y las débiles habilidades de comunicación interpersonal entre las personas que trabajan en salud son factores que contribuyen a la falta de confianza. Kasstan^48^ insta a las instituciones de salud pública a mejorar la confianza del público en la vacunación infantil, en lugar de recurrir a políticas de vacunación obligatorias y coercitivas para mejorar la baja cobertura de vacunación.

#### Leslie

Esta madre indicó que estaba muy agradecida de poder finalmente acceder a la vacuna covid-19 para su hijo de tres años. Las dos respuestas principales a este comentario, que generaron 382 reacciones de “me gusta”, sugirieron que no debería sentirse agradecida. Un usuario argumentó que Leslie está “matando” a sus hijos con la decisión de recibir la vacuna contra el covid-19, mientras que muchos otros comentaron con variaciones de “pobre bebé” y “qué vergüenza”. Otros juzgaron a Leslie por ser negligente y darles a sus hijos algo de lo que ella no sabe nada.

El ejemplo de Leslie y su hijo pequeño muestra cómo la vacunación (quién decide, recibe y es responsable de las vacunas) es un proceso de género. La toma de decisiones sobre vacunas es parte de un proceso que supone una carga desproporcionada para las mujeres y las madres^16^. Como resultado, se culpa fervientemente o se felicita a las madres por las decisiones que toman en materia de vacunas con respecto a sus hijos. Este proceso es una extensión de cómo las mujeres soportan una carga mayor con respecto a la vacunación en la sociedad. Por ejemplo, Siu *et al*.^50^ encontraron que la vacuna contra el virus del papiloma humano (VPH) está “feminizada” y “moralizada” en el sistema de valores patriarcal, lo que impone aún más la carga de enfermedad a las mujeres. En el caso de la vacuna contra el VPH, los hombres no han sido el objetivo de los mensajes de salud pública en muchos países del mundo, a pesar de ser portadores potenciales de la enfermedad. Es decir, en muchos contextos globales, los esfuerzos se han dirigido a vacunar a las mujeres (aquellas que probablemente desarrollen síntomas graves o mortales, es decir, cáncer de cuello uterino), pero no a sus parejas sexuales masculinas, que podrían infectarlas. El hecho de que los hombres no hayan sido el objetivo de crear un amortiguador protector que limite la propagación del VPH a las mujeres es indicativo de los valores sociales. Charles^13^ sostiene además que el fenómeno claramente sexista de la vacilación ante las vacunas se cruza con los legados coloniales. En su etnografía centrada en las dudas sobre las vacunas en Barbados, Charles^13^ ofrece un análisis tecnocientífico feminista crítico de la biopolítica poscolonial.

#### Gabi

Gabi indicó que el pediatra de sus hijos le dijo que la vacuna contra el covid-19 es un “no” definitivo, porque no es segura. Con esta guía, ella definitivamente decidió que no dejarría que su hijo sea vacunado. En respuesta, Becca indicó que su médico también le recomendó que no les aplicara a sus hijos la vacuna Gardasil cuando estuvo disponible y que está muy agradecida por tener un médico honesto. Otras personas felicitaron a Gabi y Becca en los comentarios por encontrar médicos que obedezcan su juramento hipocrático de no hacer daño.

Madres como Gabi y Becca son conscientes de los conflictos de intereses financieros entre los pediatras. Las aseguradoras “basadas en el valor” incentivan a los pediatras con pagos de bonificación para que aconsejen a padres y madres que sigan el calendario de vacunas recomendado por los CDC y administren cada inyección a sus pacientes. Por ejemplo, la compañía Blue Cross Blue Shield of Michigan sigue un modelo de pago por desempeño, en el que los proveedores reciben $400 dólares por cada niño de dos años que haya recibido las veinticuatro a veinticinco vacunas, si el proveedor también ha administrado todas las vacunas a al menos el 63% de sus pacientes. Los conflictos de intereses financieros también se extienden a la industria farmacéutica. Al señalar explícitamente estos conflictos de intereses, un usuario de Facebook comentó sarcásticamente que Pfizer nunca mentiría sobre la seguridad de la vacuna covid-19 para niños pequeños.

En términos más generales, los ejemplos de Gabi y Becca pueden ser abordados desde la perspectiva de Elżbieta Grodzicka^51^, quien examina la teorización de la conspiración sobre vacunas como una cuestión relacional que involucra a investigadores, formuladores de políticas, profesionales médicos, pacientes y sus familias, administradores de salud e industrias farmacéuticas. Grodzicka escribe: “Este enfoque, en lugar de centrar nuestra atención únicamente en los demás (aquellos que apoyan la vacilación, el arrepentimiento o la incredulidad en materia de vacunación), requiere que también prestemos atención a quienes están implicados en esos conflictos y tal vez estén más cerca de nosotros”^51^. Kirsten Hastrup enfatiza que el conocimiento es una cuestión relacional que surge entre personas en un campo dialógico^52^.

### Lógicas contrapuestas que subyacen a las actitudes provacunación, antivacunación y vacilación ante las vacunas contra el covid-19

Entre las dieciocho mujeres que observamos en el grupo focal en línea, once dijeron que planeaban administrar la vacuna contra el covid-19 a su hijo pequeño, mientras que tres dijeron que no y cuatro dudaron. Ahora destacamos las lógicas provacunación, antivacunación y de vacilación ante las vacunas entre las mujeres participantes del grupo focal.

#### Lógicas provacunación

*Los padres deben administrar a sus hijos todas las vacunas pediátricas recomendadas tan pronto como estén disponibles y sean accesibles*

Ejemplo: Tanya, una madre en el foro en línea, indicó que su hijo recibió la vacuna tan pronto como fue elegible. Sylvia coincidió en que era importante que su hijo recibiera todas las vacunas disponibles. Cuando se le preguntó por qué le estaba dando a su hijo la vacuna contra el covid-19, Liz respondió que su hijo había recibido todas las vacunas infantiles recomendadas. Las madres del grupo focal enfatizaron específicamente la seguridad y la necesidad de las vacunas infantiles contra enfermedades infecciosas como las paperas, la polio y el sarampión, al argumentar que la vacuna covid-19 era segura y necesaria para niños y niñas. Según su razonamiento, las madres no debían dudar en administrar la vacuna contra el covid-19 a sus hijos si permitían que recibieran otras vacunas.

*Las vacunas, incluida la vacuna contra el covid-19, son seguras*

Ejemplo: Roxana defendió la seguridad de las vacunas contra el covid-19 y la seguridad de las vacunas en general.

*Aunque es posible que la vacunación no elimine la posibilidad de contraer covid-19, los niños pequeños deben vacunarse para protegerse a sí mismos y a los demás de los peores síntomas*

Ejemplo: Varias madres en el foro en línea enfatizaron la reducción de los síntomas de quienes contraían covid-19 después de haber sido vacunados contra la enfermedad. Los comentarios mostraban cómo algunas familias valoraban la vacunación contra el covid-19 para niños y niñas pequeños porque proporcionaba un amortiguador protector que limitaba la propagación del covid-19 a otros miembros del hogar o la comunidad. Por ejemplo, una madre reconoció que su hijo no corría riesgo de desarrollar un caso grave de covid-19; sin embargo, decidió vacunar a su hijo para “hacer su parte” y proteger a otras personas que pudieran estar en riesgo. En este sentido, Ramírez y Mackey^53^, al consideran el papel de las relaciones inmediatas en la toma de decisiones sobre vacunación, muestran que los amigos y familiares suelen ejercer la mayor influencia sobre la toma de decisiones sobre vacunas. En ocasiones, las personas que se muestran reacios a vacunarse y viven en hogares multigeneracionales pueden sentirse presionadas a vacunarse cuando tienen en cuenta a otros miembros de la familia que corren un mayor riesgo de desarrollar síntomas potencialmente mortales; es decir, aunque las personas tal vez no quieran vacunas, sienten que deben vacunarse para proteger o complacer a sus seres queridos en casa.

#### Lógicas antivacunas

*La madre es inmutable, lo que indica que nada la convencería de vacunar a su hijo*

Ejemplo: Jane, una madre del foro en línea indicó que su hijo no sería vacunado contra el covid-19 y no estaba dispuesta a discutir al respecto.

*La vacunación contra la covid-19 no elimina la transmisión de enfermedades y, por tanto, no vale la pena*

Ejemplo: Cristina escribió en el foro en línea que, según un comunicado de los CDC, la vacuna contra el covid-19 no evitaba que las personas contrajeran o transmitieran la enfermedad. Si bien su afirmación era cierta, en el sentido de que ninguna vacuna puede eliminar totalmente la transmisión de enfermedades, un comunicado de prensa de los CDC del 7 de junio de 2021 indicó que las vacunas contra el covid-19 reducen el riesgo de infección para las personas completamente vacunadas y hacen que la enfermedad sea más leve y más breve en personas vacunadas que contraen covid-19.

### Lógicas de vacilación ante las vacunas

*Las dudas sobre las vacunas se deben al proceso de aprobación acelerado de la FDA y a la falta de información disponible sobre el uso pediátrico de las vacunas covid-19. No se han recopilado suficientes datos para demostrar la seguridad de la vacuna covid-19 para niños pequeños. En lugar de actuar apresuradamente y tomar una decisión equivocada, tiene más sentido esperar a tener suficiente información.*

Ejemplo: Laura publicó en el foro en línea que no confiaba en la seguridad de la vacuna contra el covid-19 “todavía”. Abigail indicó que estaba esperando, porque darle a su hijo la vacuna contra el covid-19 era una acción que podía elegir hacer más adelante, pero que no podía deshacer. Otras madres coincidieron en que debido al proceso de aprobación acelerado de la vacuna contra el covid-19, esta vacuna no debía compararse con otras vacunas infantiles. En lugar de rechazar rotundamente las vacunas, las personas que tienen cierto grado de escepticismo sobre las vacunas pueden ser selectivas sobre las vacunas que deciden aceptar^47^. Sobo *et al*.^54^ sugieren que las posiciones múltiples, a veces contradictorias, de los vacunadores selectivos sobre la vacunación son colecciones ensambladas que reflejan las prácticas contemporáneas de curación para filtrar la información. Estas prácticas de curación incluyen narrativas de “colmena” digitales experimentadas y construidas colectivamente que, según Goldberg^43^ son ​​un síntoma, no una causa, de la desconfianza pública en las instituciones científicas. Mientras padres y madres que vacunan selectivamente adoptan una visión curatorial hacia la información, los consumidores de atención médica comprometidos subrayan la necesidad de un enfoque no categórico que reconozca la naturaleza fluida y polivalente del razonamiento sobre las vacunas.

Nuestros hallazgos sugieren que los padres pueden sentirse más seguros a la hora de administrar la vacuna covid-19 a sus hijos pequeños si tuvieran acceso a datos sólidos que respalden los beneficios. Según la literatura existente, las actitudes de los individuos están influenciadas por los constantes cambios en los niveles de confianza del público en torno a las vacunas. Larson y Broniatowski^12^ documentan una alta volatilidad en la confianza en las vacunas en el contexto de la covid-19. Sugieren que los niveles de confianza en las vacunas están influenciados por los altibajos de los aumentos de virus, así como por la (des)información adicional sobre las vacunas. Al describir la vacuna contra el virus de la influenza H1N1 durante la pandemia de 2009, Danielle Ofri llamó “epidemiología emocional” al pasaje del sentimiento caracterizada por la ansiedad por la vacuna, luego la vacilación y posteriormente el rechazo a recibir la vacuna^55^.

*Las experiencias personales con la vacuna covid-19 y el historial médico de cada niño o niña influyen en las decisiones de vacunación*

Ejemplo: Después de observar y experimentar infecciones irruptivas, algunas de las madres en el grupo focal en línea expresaron su insatisfacción con la eficacia de la vacuna covid-19. Por tanto, se mostraron reacios a vacunar a sus hijos. Otras madres basaron sus decisiones en los antecedentes médicos de sus hijos individuales, por ejemplo, si el niño tenía asma y cómo había respondido a otras vacunas en el pasado.

Padres y madres, en su decisión, consideran la biología de cada niño, el tamaño, la susceptibilidad a enfermedades específicas, los peligros ambientales, las condiciones médicas y de salud^54^ y sus propias experiencias individuales preexistentes de iatrogénesis^47^^,^^53^. El género es una lente importante para examinar cómo las mujeres de diferentes orígenes raciales y étnicos traducen las experiencias personales de iatrogénesis en dudas sobre las vacunas para sus hijos^47^. Además, dado que las madres suelen desempeñar el papel más importante en la toma de decisiones médicas para sus hijos pequeños, estas experiencias de iatrogénesis son evidentes en el calendario de vacunas que las madres eligen seguir para sus hijos.

Brunson y Sobo^56^ descubrieron que en California y el estado de Washington, en lugar de fusionarse en marcadas polaridades, las percepciones de padres y madres sobre la vacunación infantil eran conjuntos diversos, dinámicos y multidimensionales. Asimismo, Dubé *et al*.^15^ afirman que las decisiones de los padres de utilizar los servicios de vacunación son multifactoriales, heterogéneas y complejas. Los procesos de evaluación y gestión de riesgos relacionados con las vacunas son altamente individualizados y pueden estar influenciados por ideologías neoliberales^16^.

*Las madres reticentes a las vacunas no muestran una desconfianza total en las instituciones científicas*

Ejemplo: En contraste con la descripción pública de que las dudas sobre las vacunas se deben a la ignorancia o la falta de información, las madres que dudan sobre las vacunas en nuestro grupo focal participaron en una “reflexión activa”, buscando información de lo que consideran fuentes confiables y tomando decisiones responsables e informadas. Basadas ​​en la percepción de riesgo y en juicios de valor basados en sus propias vidas^57^, buscaron activamente información de sus pediatras, pero no la aceptaron incondicionalmente y sin reservas, sino que la combinaron con “su propia investigación”. Es decir, integraron información de la institución médica con información de lo que consideran otras fuentes confiables. Como resultado de este proceso, algunas madres enfatizaron el riesgo de efectos a largo plazo de la infección por covid-19 en los niños pequeños, mientras que otras enfatizaron los riesgos asociados con la vacuna covid-19. Nuestros hallazgos señalan la importancia de que los proveedores se asocien con padres y madres y participen en la toma de decisiones conjunta con respecto a las vacunas infantiles.

*Los padres a veces pueden encontrar información contradictoria en entornos médicos*

Ejemplo: Una madre, Elvia, proporcionó un enlace a un artículo publicado por una facultad de medicina como prueba de que las vacunas contra el covid-19 no tendrían efectos secundarios a largo plazo. Por otro lado, Patricia, una madre en el grupo focal en línea, contó cómo su pediatra le dijo que esperara para vacunarse contra el covid-19 ya que la vacuna posiblemente podría dejar infértiles a sus hijas.

Padres y madres se enfrentan a información contradictoria en la esfera pública, una situación que disminuye la credibilidad y se enfrentan al desafío de decidir en qué fuentes de información confiar. Al mismo tiempo, las creencias sobre las vacunas influyen en la forma en que padres y madres interpretan los resultados de la vacunación. Por ejemplo, Rozbroj *et al*.^14^ documentaron informes de lesiones infantiles permanentes, relacionadas con las vacunas por parte de padres que creían que algunas o todas las vacunas debían rechazarse. Al mismo tiempo, las creencias sobre las vacunas y el autismo pueden no ser tan extremas como a menudo se presentan en los medios de comunicación. En un estudio realizado por Anderson-Chavarria y Turner, 32 de 35 padres y madres entrevistados creían que el autismo es el resultado de riesgos genéticos desencadenados por un factor ambiental^58^. Los desencadenantes incluyen diversos contaminantes ambientales y vacunas; es decir, las vacunas se consideran uno de los muchos posibles “desencadenantes” de un niño genéticamente predispuesto al autismo. Si bien los padres o las madres no percibieron que las vacunas eran universales, las dudas sobre las vacunas pueden ser indicativas de una falta de confianza del público en la ciencia.

Majid y Ahmad^49^ señalan cómo los padres confían en la información de los proveedores de medicina complementaria y alternativa cuando consideran que la información de los proveedores de medicina alopática es insuficiente, ya que sienten que es más fácil tener una discusión abierta y honesta con los proveedores de medicina complementaria y alternativa, en comparación con los médicos alópatas^15^^,^^54^.

## DISCUSIÓN

### Lógicas en competencia y razonamientos polivalentes respecto de las vacunas

Los profesionales médicos a menudo asumen que brindar a las personas la información correcta sobre la seguridad de las vacunas y los riesgos de no vacunarlas las llevará a tomar la decisión responsable de vacunarse^11^^,^^59^^,^^60^^,^^61^. Sin embargo, Roberts y Mitchell^11^ revelan que las personas que dudan en vacunarse toman en serio su responsabilidad de tomar buenas decisiones de salud, buscan asesoramiento médico, incorporan y evalúan información médica sobre la vacunación y consideran cuidadosamente los riesgos y beneficios de vacunarse. Sobo *et al*.^41^ muestran que padres y madres selectivos, que no vacunaban, mostraban el tipo de participación autoinformada recomendada por el sistema de salud. En su estudio, Rozbroj *et al*.^14^ manifiestan que tener hijos impulsó a padres y madres a aprender más sobre las opciones de vacunas. De manera similar, Ward *et al*.^62^ señalan que tanto las familias que rechazan las vacunas como las que dudan participan en una búsqueda continua de información sobre cómo tomar las mejores decisiones médicas para sus hijos e hijas, lo que llevó a muchos a cuestionar o desconfiar de la epistemología médica occidental.

Estos académicos se basan en la reconceptualización de Leach y Fairhead^8^ sobre la vacilación ante las vacunas, que se aleja del “modelo de déficit” convencional (es decir, la vacilación ante las vacunas es el resultado de la falta de conocimiento, confianza o racionalidad de un individuo^43^^,^^51^ y hacia una comprensión de la vacilación ante las vacunas como una “reflexión activa” (es decir, un pensamiento positivo y comprometido sobre cómo cuidar mejor de uno mismo y de los demás). Este replanteamiento cambia el enfoque de por qué algunas personas no se vacunan a por qué la vacunación no coincide con el deseo de salud de las personas^8^.

En muchos casos, las familias que dudan en vacunarse son especialmente proactivos cuando se trata de la salud de sus hijos e hijas. La “crianza salutogénica”, término utilizado por Ward *et al*.^62^, describe cómo padres y madres practican actividades que promueven la salud para estimular la inmunidad natural y protegerlos de enfermedades. Las prácticas parentales salutogénicas incluyen amamantar, cocinar desde cero, comer alimentos orgánicos y/o de cosecha propia y reducir la exposición a conservantes y toxinas. Estas prácticas se superponen con la “maternidad intensiva” -generalmente asociada con madres blancas y neoliberales- que se relaciona con el escepticismo sobre las vacunas^47^. Padres y madres creen que a través de estas prácticas pueden reducir o anular la necesidad de vacunas. Ward *et al*.^62^ enmarcan la crianza salutogénica como una “lógica de cuidado” que las familias consideran internamente consistente, lógicamente interrelacionada e interdependiente.

Majid y Ahmad^49^ ofrecen la metáfora de un tren de engranajes para comprender las superposiciones y relaciones entre diversos factores que refuerzan la vacilación, el rechazo y el retraso en las vacunas. En su metáfora, cada engranaje representa uno de siete factores: vida “natural” y “orgánica”, lo que Ward *et al*.^62^ llama “crianza salutogénica”; percepciones de otros padres; experiencias previas; fuentes de información, desafíos y preferencias; políticas de vacunas obligatorias; desconfianza en los actores del sistema de salud y experiencias de interacción con los proveedores de atención de salud. Si bien estos engranajes funcionan al unísono para impulsar las dudas sobre las vacunas, la toma de decisiones compartida es un engranaje que puede revertir el mecanismo girando en la dirección opuesta.

Rozbroj *et al*.^14^ indican que tener hijos obliga a padres y madres a examinar sus creencias sobre las vacunas, especialmente quienes dudan o se niegan a vacunarse. Las familias a menudo no “aceptan” o “rechazan la vacuna” del todo, sino que consideran las vacunas individualmente, teniendo en cuenta también la biología, el tamaño, la susceptibilidad a las enfermedades de los niños^41^. Para algunos, no vacunarse puede ser una decisión responsable e informada que sea “adecuada para ellos” basada en la percepción del riesgo y los juicios de valor en el contexto de sus vidas^57^. En el caso de la vacunación infantil, los padres clasifican la información relacionada con la vacuna de manera “irregular” y dependiente del contexto. Esto es especialmente relevante en el caso de la vacuna contra el covid-19, ya que las dudas sobre la vacuna pueden deberse al proceso de aprobación acelerado de la FDA y a la falta de información sobre las vacunas.

## CONCLUSIONES

Si bien la prensa médica y no especializada caracteriza la vacunación como una controversia polarizadora, la investigación cualitativa revela una multitud de perspectivas sobre el tema. Los resultados de este estudio resaltan la necesidad de matizar la forma en que se abordan, comprenden y definen la aceptación, el rechazo y la vacilación de las vacunas. Se puede considerar que la aceptación, el rechazo y la vacilación de la vacuna operan en un continuo en lugar de desarrollarse en categorías discretas. Sorrell y Butler^42^ señalan que: “Las posiciones que alguna vez estuvieron asociadas con posturas religiosas o conservadoras tradicionales han dado paso a puntos de vista muy dispares que trascienden las divisiones tradicionales izquierda/derecha/religiosas”^42^. En la “zona gris” entre los bandos pro y antivacunas, las personas que dudan sobre las vacunas van desde “escépticos” hasta “indecisos” o “vacilantes”^9^.

En nuestro grupo focal en línea, las madres demostraron un razonamiento fluido y polivalente sobre la vacuna con respecto a la vacuna covid-19. Estas diferentes líneas de razonamiento fueron presentadas por madres que permitieron que sus hijos recibieran vacunas infantiles, y ambas líneas de razonamiento se basaron en datos científicos disponibles (por ejemplo, comunicados de prensa de los CDC, resúmenes de estudios de investigación escritos para una audiencia pública e infografías o artículos publicados por facultades de medicina, etc.) e información anecdótica (por ejemplo, niños y niñas de la comunidad que contraían un caso grave de covid-19, se recuperaban rápidamente de un caso leve de covid-19, experimentaban una infección irruptiva a pesar de estar completamente vacunados o tenían una reacción a la vacuna contra el covid-19). Este ejemplo ilustra cómo la información se puede interpretar de manera diferente según las prácticas curatoriales de padres y madres.

En última instancia, las familias valoran diferentes fuentes de información en función de su propia percepción de la confiabilidad de la información y priorizan la información que está alineada con sus creencias personales^54^. En el grupo focal en línea, Tiffany, una madre a favor de la vacunación, respondió a Roxana, una madre reticente a las vacunas con quien no estaba de acuerdo, diciendo que Roxana debería simplemente decir que no quería darle la vacuna covid-19 a su hijo en lugar de difundir narrativas falsas. Mi interpretación del intercambio, sin embargo, no es que Roxana estuviera intentando difundir narrativas falsas, sino que sus criterios para lo que califica como información confiable diferían significativamente de los criterios de Tiffany. De manera similar, Elvia respondió a Patricia denunciando el “mito” de la infertilidad como “información errónea utilizada para asustar a la gente” y argumentó que no hay riesgo. Stephanie refutó que el médico le haya señalado a Patricia el riesgo potencial de infertilidad. Las cinco madres priorizaban la información que estaba alineada con sus creencias, experiencias y observaciones personales del mundo que las rodeaba.

Los hallazgos de este estudio enfatizan las diferentes nociones de “riesgo” entre las madres de niños y niñas de 6 meses a cuatro años. En el grupo focal en línea que observamos, algunas madres enfatizaron los efectos desconocidos a largo plazo de la infección por covid-19 en niños y niñas pequeños, mientras que otras madres enfatizaron los efectos desconocidos a largo plazo de la vacuna contra el covid-19 (a veces combinados con el riesgo conocido de covid-19; por ejemplo, cuando los niños ya habían sido infectados con covid-19 y se habían recuperado bien de un caso leve).

Para algunas personas, renunciar a una vacuna recomendada se considera “riesgoso”; para otras, aceptar la vacuna se considera “riesgoso”. Visto a través de esta lente, no es que las personas actúen descuidadamente, sino más bien que individuos con diferentes perspectivas intentan minimizar su exposición al riesgo basándose en su comprensión de lo que es riesgoso. Este enfoque hacia la aceptación, el rechazo y la vacilación de las vacunas resiste la “otredad” que está omnipresente en los debates en curso a favor y en contra de las vacunas. En última instancia, los hallazgos de este estudio y de estudios anteriores indican que una multitud de factores contribuyen a las dudas sobre las vacunas entre los padres y las madres individuales. Determinar, y no descartar, los factores subyacentes en cada caso deben hacerse con una cuidadosa consideración.
