# Supplementary material for: Vacilación ante la vacuna contra el covid-19 en Estados Unidos de América: un estudio etnográfico digital
Source: Salud Colect. 2024 Mar 26;20:e4541. [Article in Spanish] doi: 10.18294/sc.2024.4541 (PMC11822896; doi:10.18294/sc.2024.4541)
Supplement: Translation

# Translation: Hesitation towards the COVID-19 vaccine in the United States: a digital ethnographic study

**Authors:** Rosalynn Adeline Vega

**Affiliations:** 1 PhD in Medical Anthropology. Associate Professor, University of Texas Rio Grande Valley, Edinburg, USA. Rosalynn.Vega@utrgv.edu

**Keywords:** Vaccines, Child, Parents, Pediatricians, United State of America.

## Abstract

Following the authorization the use of COVID-19 vaccines in babies age six months through children four years old in the United States, some individuals (parents, pediatricians, and communicators) framed COVID-19 vaccination as an issue of access, while many others expressed hesitancy and some resisted recommendations from the US Centers for Disease Control and Prevention. In this context, this study aimed to explore: 1) divergent reactions to the authorization of COVID-19 vaccine use in children aged six months to four years; and 2) opposing logics underlying attitudes towards pro-vaccination, anti-vaccination, and vaccine hesitancy regarding COVID-19 vaccines. To achieve this, a digital ethnography was conducted, involving monitoring of 5,700 reactions to a series of eight infographics published on social media by the John Hopkins Bloomberg School of Public Health, and participant observation in an online focus group over a one-year period, from December 2021 to December 2022, consisting of 18 mothers. The findings suggest that healthcare professionals should consider different notions of “risk” when interacting with patients, especially those who are hesitant to vaccinate.

## PRESENTATION OF THE TOPIC

On June 17, 2022, the US Food & Drug Administration (FDA) authorized the use of Moderna and Pfizer-BioNTech COVID-19 vaccines in children aged 6 months and older.^1^ The following day, on June 18, the director of the Centers for Disease Control and Prevention (CDC), Rochelle P. Walensky, endorsed the Advisory Committee on Immunization Practices’ (ACIP) recommendation that all children aged 6 months to 4 years receive the COVID-19 vaccine.^2^ Leading up to the FDA authorization, some parents, pediatricians, and media outlets framed COVID-19 vaccination as an issue of access.^3^ However, studies failed to demonstrate that vaccinating infants and very young children significantly reduces severe infection cases (i.e., cases resulting in hospitalization and death).

The FDA’s briefing document of June 15, 2022 states: “In older pediatric age groups, the vaccine has been shown to prevent hospitalization and other severe sequelae [...] implementation of the vaccine for use in children 6 months to 4 years of age is likely to have a beneficial effect on morbidity and mortality associated with COVID-19 in this age group”.^4^^)^ This FDA document infers a probable benefit for young children, based on the demonstrated benefit for older pediatric age groups; however, it presents no data demonstrating vaccination’s benefit for children aged 6 months to 4 years.

Two months after FDA approval, Anne Hause et al.’s article focused on post-authorization safety of the primary series of COVID-19 vaccine in young children, through reviewing adverse events and health impacts following COVID-19 vaccination.^5^^)^ Out of 5,011 children aged 6 months to 5 years included in the study, 1,017 adverse events were reported. While this article indicates that 98.1% of the events were classified as non-serious and 1.9% as serious (meaning reactions after vaccination are expected, but serious adverse events are rare), the study does not investigate whether vaccination has a beneficial effect on morbidity and mortality. If COVID-19 vaccination has not been demonstrated to be beneficial for young children, instead of “process of thinking,” one could use “reasoning” or “way of thinking.” Therefore, the phrase could be: What are the concerns, values, and reasoning of parents in favor of vaccination?

On the other hand, doubts about vaccines are becoming increasingly common in the US. After the approval of the COVID-19 vaccine for young children^1^ on June 18, 2022, many parents were hesitant, and some resisted the CDC’s recommendation. In fact, on November 21, 2022, according to an infographic published by the John Hopkins Bloomberg School of Public Health,^6^ only 9% of eligible children had received at least one dose of the vaccine. In another infographic published on the same day by the same institution, it reads: “Why aren’t kids under 5 getting vaccinated for Covid?.”^7^ The question that arises is: What factors lead parents to hesitate when it comes to vaccinating their children?

### Pro-vaccination, anti-vaccine, and vaccine hesitancy

The term pro-vaccination describes the “silent majority” that considers vaccination as an unquestionable public good.^8^ Kashyap et al.^9^ characterize this group as those who readily accept vaccination due to implicit trust in the government, its healthcare system, and public schools. Vaccines are widely associated with technological progress, modern medicine, and cost-effective public health care.

According to Kashyap et al.,^9^ the anti-vaccine movement is constituted by a small but vocal minority of so-called “challengers” who share a worldview and medical ideology that disregards vaccination as an antidote. Vogel^10^ describes this group as the 2% who vehemently reject vaccines and cannot be persuaded otherwise. The anti-vaccine movement has been characterized as a war between pediatricians and patients.^11^ Anti-vaccine parents often subscribe to alternative scientific philosophies or hold conspiratorial beliefs reflecting a deeply ingrained distrust in the government (e.g., vaccines are designed to reduce minority populations, render children powerless or infertile, and benefit the commercial interests of pharmaceutical companies). In the context of the COVID-19 pandemic, organized anti-vaccine groups argued that COVID-19 was not dangerous, but the vaccine was, and that vaccine advocates could not be trusted.^12^

Regarding vaccine hesitancy, an increasing number of individuals question the necessity and safety of vaccines. Charles^13^ points out that vaccine skepticism is distinct from rejection and also diverges from anti-vaccine beliefs. Larson and Broniatowski^12^ indicate that vaccine-hesitant individuals are those who are undecided. Although hesitancy about potential vaccine safety risks is not the same as being against vaccines, people who hesitate to vaccinate risk being stigmatized as “anti-vaxxers” by healthcare professionals.

Indeed, Rozbroj et al.^14^ note that many parents who hesitate to vaccinate their children are concerned about vaccines regarding the CDC-recommended schedule (for example, the administration of the hepatitis B vaccine in infants, despite hepatitis B being transmitted through blood, semen, or other bodily fluids via sexual contact, intravenous drug use, etc.). These individuals may simply delay receiving a vaccine due to safety concerns, only to ultimately receive it. Although most hesitant mothers ultimately decide to follow the recommended vaccination schedule for their children, they still feel ambivalent about their decision.^15^

Vaccination rates are declining, and outbreaks of preventable diseases have increased in some areas of the US. In 2015, a measles outbreak originating in Disneyland, California spread to 6 other states in the US, as well as to Mexico and Canada.^16^ That same year, California passed Senate Bill 277, which eliminated the personal belief exemption for vaccines when entering school. By doing so, California became the third state in the US to eliminate non-medical exemptions.

More recently, increasing doubts about COVID-19 vaccines have led to declines in vaccination coverage across the US.^17^ Williams and O’Leary estimate that the vaccination coverage threshold necessary to prevent outbreaks such as measles is 95%. During the 2021-2022 school year, vaccination coverage for measles, mumps, and rubella among kindergarten children dropped to 93%, the lowest in a decade.^17^ The District of Columbia had less than 90% coverage. Globally, the rise in vaccine hesitancy among both developed and developing countries has led the World Health Organization to declare declining vaccination as one of the top ten threats to global health.^18^

While many parents readily accepted the COVID-19 vaccine for their young children, a considerable minority refused or delayed acceptance of the vaccine. Faced with this scenario, this article aims to address the following dimensions: 1) divergent reactions to the authorization of COVID-19 vaccine use in children aged 6 months to 4 years; and 2) contrasting logics underlying pro-vaccination, anti-vaccination, and vaccine-hesitant attitudes toward COVID-19 vaccines.

## METHODOLOGY

To explore the attitudes of parents regarding pediatric COVID-19 vaccines, we conducted a virtual ethnography, which Hine defines as ethnography conducted “in, of, and through the virtual.”^19^ Ethnographies increasingly study practices, themes, groups, and modes of communication that rely entirely on digital technologies for their existence.^20^^,^^21^^,^^22^^,^^23^^,^^24^^,^^25^^,^^26^^,^^27^^)^ Although virtual ethnography entails an “experiential rather than physical displacement,”^28^ the key ethnographic principle is to develop an understanding of the social phenomena in question through participant observation (i.e., immersion) and progressive data collection (e.g., systematic research), which remains consistent with traditional ethnography.^19^ Furthermore, while the disembodiment of digital interactions may make the online world seem like a “non-space,”^29^ ethnographic research demonstrates how social collectives inhabit digital media as a “cultural location” through new modes of communication.^30^^,^^31^ Therefore, ethnography can be leveraged as a valuable tool for analyzing complex online social communities.

On one hand, in the days following the FDA’s authorization of Moderna and Pfizer-BioNTech COVID-19 vaccines for children, we monitored a total of 5,700 reactions to a series of eight infographics published on social media by the Johns Hopkins Bloomberg School of Public Health regarding the June 18, 2022 authorization of the COVID-19 vaccine for children aged 6 months to 4 years. While the online focus group we created allowed for intimate person-to-person discussion, the analysis of reactions on social media to the infographics captures attitudes from a larger sample, which Airoldi^32^ might term a “metacamp”.

Additionally, social media analysis provides valuable insights, as social media practices often reflect how participants in ethnographic research navigate the broader social and material world.^33^ On the other hand, we conducted participant observation in an online forum through the Peanut app, designed for mothers raising their children during the COVID-19 pandemic. While the Peanut app is available to Android and iOS users worldwide, the vast majority of women reside in the US. We created a thread asking if participants were planning to vaccinate their children against COVID-19 and why, thus creating an online focus group. In doing so, we created a focal object (i.e., parents’ attitudes toward COVID-19 vaccination for children aged 6 months to 4 years) within a relatively stable “contextual” field (i.e., the Peanut app’s online forum, which Airoldi^32^ and Caliandro^34^ refer to as a “classic” online community). In this thread, 18 individuals discussed what motivates them to seek the COVID-19 vaccine for their young children or, alternatively, why they resisted the recent CDC recommendation. Participants not only provided their personal response to the question, but many engaged with each other in an ongoing debate on the topic. The 45 thread posts were included as data points for this study.

We opted for “active” participation in this group (i.e., creating a thread within the group, thereby constituting an online focus group) rather than discrete online “lurking.” Hine^28^ notes that discrete virtual ethnography (i.e., observing online interactions without attempting to interact with members) is considerably appealing to some ethnographers as it provides a means to study social life as it is lived; however, it also presents ethical issues.^35^ Steinmetz^36^ even argues that when a researcher “lurks” in an online forum without participating, they are not conducting primary ethnographic data but rather secondary content analysis. Instead of ethnographic research, “lurking” is best described as archival research. In contrast, active participation in a group helps virtual ethnographers gain experiential knowledge about group interactions while also opening up other forms of online interaction between group participants and the ethnographer, which would be missed if ethnography were limited to discreet observation of public transmissions.

Our decision to pursue “active” participation reflects our commitment to ethics in online ethnography and our efforts to build trust with participants. According to Hine,^28^ trust is developed when the ethnographer’s presence is acceptable to members of the online social community. At the same time, virtual ethnographers can determine whether what they are observing is authentic (as opposed to mere performance for their benefit) through continuous presence in the online group. Although the data included in this article are limited to the focus group we created, our participant observation in the online forum lasted for a year. This continuous presence allowed us to build trust with the participants and determine the authenticity of the interactions we observed. Our “active” participation was made explicit to the group, along with the research characteristics. Participants were informed that their interactions would be taken as research material and that anonymity would be safeguarded at all times.

Virtual ethnographers must understand how to analyze the social and material life of digital media, given the ephemeral, mutable, hypermobile, and anonymous nature of digital data, such as internet memes, chats, social media posts, and accompanying comments.^37^^,^^38^ In addition to participant observation and online interviews, virtual ethnography can also include complementary techniques such as collecting screenshots, capturing chat logs, and analyzing social media posts.^39^ In this study, we collected screenshots of the discussion that unfolded in the thread we created about parents’ attitudes toward pediatric COVID-19 vaccination.

However, virtual ethnography on health-related topics raises ethical questions regarding user/patient privacy and public health, particularly concerning the use of (semi)private identifiable data. A representative from the Institutional Review Board for Human Subjects Research at the University of Texas Rio Grande Valley determined that, given the nature of the online forum, it was not necessary to submit the research protocol for review. The online forum constitutes a public online space accessible to people worldwide at no cost. Additionally, the individuals involved were anonymous, even to us. Participants identified themselves through a simple forum-specific identifier (which could be a name or an alias). No one in the forum could access identifying information such as last names and contact information (e.g., phone numbers, email addresses, and home addresses). The app does not contain a searchable user database. Despite this inherent anonymity, we are sensitive to the expectations of online informants, even when their activities occur in online public spaces.^40^ To further preserve the anonymity of those involved in the discussion, we omitted direct quotes from individuals’ comments, screenshots, and the usernames they used in the forum. All names used in this article are pseudonyms.

After observing how the debate unfolded within the focus group and monitoring reactions on social media to infographics published by the Johns Hopkins Bloomberg School of Public Health, we used an iterative open coding process to identify emerging themes. I then proceeded with a literature review on vaccine hesitancy to better understand how the recommendation for COVID-19 vaccination for young children represents a “disquieting” element in an already tumultuous landscape of pediatric vaccination.

### Divergent reactions to the authorization of COVID-19 vaccine use in children aged 6 months to 4 years

We observed mixed reactions to a series of eight infographics posted on Facebook by the Johns Hopkins Bloomberg School of Public Health regarding the June 18, 2022 authorization of the COVID-19 vaccine for children aged 6 months to 4 years. One of the online infographics cautions parents that if they contract COVID-19, their children may experience severe symptoms and short- and long-term health consequences. The infographic further indicates that while severe cases of COVID-19 among children are rare, nearly 1,500 children have died due to COVID-19 infection in the US. This infographic emphasizes short- and long-term health consequences and the 1,500 child deaths (these parts appear in bold font). This infographic is combined with others that dismiss fears that the COVID-19 vaccine may be potentially unsafe or cause side effects, labeling them as “unjustified skepticism” and “false ideas.”

While most posts from the Johns Hopkins Bloomberg School of Public Health generate fewer than one hundred reactions, the post containing infographics about COVID-19 vaccination for young children generated 5,700 reactions, 1,200 comments, and 664 shares. Of the total reactions, 34.5% “liked” the post, 34.5% were “angry,” 16.1% “loved” it, 10.4% “laughed,” 2.2% were “sad,” >1% used the emoji symbolizing “support and care” (hugging a heart), and >1% were “shocked.” Furthermore, comments on the post ranged from approval to anger and ridicule. We highlight individuals whose comments sparked heated debate and delve into the nature of those debates.

#### Sandra

The comment that generated the most reactions (2.9K reactions, including thumbs up, hearts, and laughter) was from Sandra, a mother of two young children. Sandra revealed that her family is unvaccinated and has had COVID-19 three times. Additionally, she indicated that she and her children have recovered faster each time than her mother, despite her mother being vaccinated against COVID-19. Responses to this comment were mixed. Some supported Sandra, offering examples of how the vaccine does not protect people from contracting or spreading the disease, and indicating that their personal experiences with COVID-19 were less severe than seasonal flu or “a hangover.” Others judged Sandra for transmitting the disease to other members of the community, including her elderly mother, who is at higher risk of serious health complications or even death.

Sandra and her family provide an example of how vaccination decision-making is a relational process. Vaccination decisions are influenced by relationships with others, just as relationships with others are affected by vaccination decisions.

Those who supported Sandra created cohesion within the group through their shared rejection of the COVID-19 vaccine. In this vein, Elisa Sobo^41^ highlights how productive vaccine rejection is for immediate social relationships. This author argues that rejection not only denies exogroup definitions but also reaffirms intragroup frameworks and, therefore, their bonds. Similarly, Charles^13^ indicates that vaccine suspicion generates relationships and affections.

Those who criticized Sandra for her decision not to vaccinate, blaming her for spreading COVID-19 to other community members and Sandra’s mother, echoed an underlying theme in one of the analyzed infographics depicting a germ tornado approaching a house. The infographic explains that COVID-19 infection is harmful to the household as it can spread among family members and that vaccinating young children can provide “peace of mind” to the family. This framework points to children as biological citizens who can play a role in preventing further spread of COVID-19 in the adult population. That is, this shift reframes the conception of children who are no longer just recipients of care but citizens responsible for caring for others.

#### Anya

The comment that garnered the second-highest number of reactions (1.7K reactions, including thumbs up, laughter, and tears) was from Anya, a mother who received her COVID-19 booster shot during pregnancy and believes it’s important to keep her children fully immunized. Her entire family of 5 members has never tested positive for COVID-19 or other viruses, and her children will continue to receive all recommended vaccines. Meanwhile, another mother named Elise responded to Anya by offering contradictory evidence to Anya’s experience: no one in her family of 6 members has been vaccinated, and no one has contracted COVID-19.

Other users left ironic comments wishing Anya’s family well with the illnesses they will develop from getting the vaccines. A woman named Rosa responded by telling Anya that she will regret her decision in the future when she discovers that her children are sterile. Another user commented that they are praying for Anya’s children to remain alive in 20 or 30 years. These comments received thumbs up, love, and laughter reactions. Others suggested that by accepting the COVID-19 vaccine for her young children, Anya would be donating her children to science and enrolling them early in a eugenics program.

Others responded that Anya would be including her children in the “intervention” group of a societal-wide clinical trial for a vaccine that has been approved without sufficient evidence. In the case of Anya’s young children, one user pointed out that by accepting the vaccine, Anya would be taking a child who has minimal risk of serious COVID-19 complications and placing them in a long-term study with unknown health consequences for their future.

Sorell and Butler^42^ argue that social media has facilitated the spread of conspiracy theories surrounding COVID-19 and mass vaccination programs. However, May Goldberg suggests in her book, *Vaccine hesitancy: Public trust, expertise, and the war on science*,^43^ that in our era of science politics, the language of science is the currency of political discourse. Therefore, debates over what scientific knowledge should be produced and how to use it replace debates over values. Perspectives on vaccination carry social weight as they address issues of sexuality, women, religion, provincial and federal health policy, and big pharma.^11^

Goldberg argues that vaccine hesitancy has been incorrectly framed as a result of a “war on science.” In this contradictory framework of vaccine hesitancy, individuals who are ignorant, scientifically illiterate, and irrational are pitted against scientific experts, or their cognitive biases sustain vaccine hesitancy in the face of evidence. However, Goldberg argues that what drives vaccine hesitancy is not an increase in anti-expert sentiment or an “epistemological populism” where everyone is their own expert and “does their own research,” but, rather, a lack of trust in scientific institutions. That is, distrust in scientific institutions may lead parents to question biomedical authority and demedicalize their health approaches.

Without transparency from scientific institutions and without public trust, science cannot effectively guide policies. In this context, strengthening vaccine credibility through increased trust in scientific institutions would be more effective than existing measures to promote vaccine coverage. So far, the medical institution has attempted to build trust by focusing on communication between patients and providers rather than addressing general public trust in childhood vaccines. Lauren Vogel^10^ advises providers to focus their efforts on building trust with vaccine-hesitant “fence-sitters.” Instead of focusing on parents who staunchly reject vaccines, Vogel suggests that providers focus on convincing those who are hesitant about vaccines to accept recommendations by providing them with information, offering reassurances, and using their own stories of encounters with vaccine-preventable diseases. This approach is guided by estimates that less than 2% of parents are “anti-vaccine,” while up to 30% are hesitant to vaccinate.^45^ Unfortunately, Vogel’s strategies for convincing hesitant individuals of CDC recommendations do not demonstrate an approach and understanding of the other, as her approach to “building trust” does not propose that providers carefully consider the reasons behind parents’ doubts and understand that vaccine hesitancy is not simply due to a lack of information.

When distrust in the government contributes to doubts about vaccines, this distrust can be attributed to historical community experiences, especially among BIPOC (Black, Indigenous, People of Color) communities.^12^^,^^46^^,^^47^ The public may not trust the scientific community due to a history of medical racism or the commodification of sciences (prioritizing profit over safety), especially medical science. Specific injustices include eugenics, quarantines of minority groups, the destruction of “unhealthy” neighborhoods inhabited by marginalized communities, the opioid crisis, and unethical research such as the Tuskegee experiment.^46^ Kasstan^48^ frames vaccine hesitancy as part of a broader shift in public health relations with minority groups. He argues that a better understanding of vaccine decision-making will help avoid harmful representations of minorities while also addressing vaccine hesitancy in a more sustainable and trustworthy manner.

#### Tilda

A mother whom we’ll refer to as Tilda recounted how the COVID-19 vaccine had been encouraged, then incentivized, and ultimately imposed on the adult population of the United States. She argued that as a result of this, parents will not willingly vaccinate their children against COVID-19. Majid and Ahmad^49^ point out how extreme pressures to vaccinate in the form of financial sanctions (i.e., not receiving government allowances for childcare) and exclusion (i.e., not being accepted in public school) have led to limited learning opportunities for children of hesitant parents. In this regard, Kashyap et al.^9^ indicate that feeling coerced by school authorities, poor relationships between parents and healthcare providers, and weak interpersonal communication skills among healthcare workers are contributing factors to the lack of trust. Kasstan^48^ urges public health institutions to build public trust in childhood vaccination instead of resorting to mandatory and coercive vaccination policies to improve low vaccination coverage.

#### Leslie

This mother indicated that she was very grateful to finally access the COVID-19 vaccine for her three-year-old son. The two main responses to this comment, which garnered 382 “likes,” suggested that she shouldn’t feel grateful. One user argued that Leslie is “killing” her children with the decision to get the COVID-19 vaccine, while many others commented with variations of “poor baby” and “shameful.” Others judged Leslie for being negligent and giving her children something she knows nothing about.

The example of Leslie and her young son demonstrates how vaccination (who decides, receives, and is responsible for vaccines) is a gendered process. Decision-making about vaccines is part of a process that disproportionately burdens women and mothers.^16^ As a result, mothers are fervently blamed or praised for the vaccine decisions they make regarding their children. This process is an extension of how women bear a greater burden regarding vaccination in society. For example, Siu et al.^50^ found that the human papillomavirus (HPV) vaccine is “feminized” and “moralized” within the patriarchal value system, further imposing the burden of disease on women. In the case of the HPV vaccine, men have not been the target of public health messages in many countries worldwide, despite being potential carriers of the disease. That is to say, in many global contexts, efforts have been directed toward vaccinating women (those likely to develop severe or deadly symptoms, such as cervical cancer), but not their male sexual partners who could infect them. The fact that men have not been targeted to create a protective buffer that limits the spread of HPV to women is indicative of societal values. Charles^13^ further argues that the clearly sexist phenomenon of vaccine hesitancy intersects with colonial legacies. In her ethnography focused on vaccine hesitancy in Barbados, Charles^13^ offers a critical feminist technoscientific analysis of postcolonial biopolitics.

#### Gabi and Becca

Gabi stated that her children’s pediatrician told her that the COVID-19 vaccine is a definite “no” because it’s not safe. With this guidance, she definitely decided not to let her child be vaccinated. In response, Becca mentioned that her doctor also advised against giving her children the Gardasil vaccine when it was available, and she is very grateful to have an honest doctor. Other people congratulated Gabi and Becca in the comments for finding doctors who adhere to their Hippocratic oath of doing no harm.

Mothers like Gabi and Becca are aware of the financial conflicts of interest among pediatricians. “Value-based” insurers incentivize pediatricians with bonus payments to advise parents to follow the vaccine schedule recommended by the CDC and administer each shot to their patients. For example, Blue Cross Blue Shield of Michigan follows a pay-for-performance model, where providers receive $400 per two-year-old child who has received 24 to 25 vaccines, if the provider has also administered all vaccines to at least 63% of their patients. Financial conflicts of interest also extend to the pharmaceutical industry. Explicitly pointing out these conflicts of interest, a Facebook user sarcastically commented that Pfizer would never lie about the safety of the COVID-19 vaccine for young children.

More broadly, the examples of Gabi and Becca can be addressed from the perspective of Elżbieta Grodzicka,^51^ who examines vaccine conspiracy theorizing as a relational issue involving researchers, policymakers, medical professionals, patients and their families, health administrators, and pharmaceutical industries. Grodzicka writes: “This approach, rather than solely focusing our attention on others (those who support vaccine hesitancy, regret, or disbelief), requires us to also pay attention to those who are involved in those conflicts and may be closer to us.” Kirsten Hastrup emphasizes that knowledge is a relational matter that arises among people in a dialogical field.^52^

### Opposing logics underlying pro-vaccination, anti-vaccination, and vaccine hesitancy attitudes towards COVID-19 vaccines

Among the eighteen women observed in the online focus group, eleven stated that they planned to administer the COVID-19 vaccine to their young child, while three said they would not, and 4 were undecided. Now, we highlight the pro-vaccination, anti-vaccination, and vaccine-hesitant logics among the women participating in the focus group.

#### Pro-vaccination logic

*Parents should administer all recommended pediatric vaccines to their children as soon as they are available and accessible.*

Example: Tanya, a mother in the online forum, indicated that her child received the vaccine as soon as he was eligible. Sylvia agreed that it was important for her child to receive all available vaccines. When asked why she was giving her child the COVID-19 vaccine, Liz responded that her child had received all recommended childhood vaccines. The mothers in the focus group specifically emphasized the safety and necessity of childhood vaccines against infectious diseases such as mumps, polio, and measles, arguing that the COVID-19 vaccine was safe and necessary for boys and girls. According to their reasoning, mothers should not hesitate to administer the COVID-19 vaccine to their children if they allowed them to receive other vaccines.

*Vaccines, including the COVID-19 vaccine, are safe.*

Example: Roxana defended the safety of COVID-19 vaccines and vaccines in general.

*While vaccination may not eliminate the possibility of contracting COVID-19, young children should be vaccinated to protect themselves and others from the worst symptoms.*

Example: Several mothers in the online forum emphasized the reduction in symptoms for those who contracted COVID-19 after being vaccinated against the disease. Comments showed how some families valued COVID-19 vaccination for young boys and girls because it provided a protective buffer that limited the spread of COVID-19 to other household or community members. For example, one mother acknowledged that her child was not at risk of developing a severe case of COVID-19; however, she decided to vaccinate her child to “do her part” and protect other people who may be at risk. In this regard, Ramírez and Mackey,^53^ considering the role of immediate relationships in vaccine decision-making, show that friends and family often exert the greatest influence on vaccine decision-making. At times, individuals who are hesitant to vaccinate and live in multigenerational households may feel pressured to get vaccinated when considering other family members who are at higher risk of developing potentially deadly symptoms; that is, while individuals may not want vaccines, they feel they must vaccinate to protect or please their loved ones at home.

#### Anti-Vaccination Logics

*The mother is steadfast, indicating that nothing would convince her to vaccinate her child.*

Example: Jane, a mother from the online forum, stated that her child would not be vaccinated against COVID-19 and she was not willing to discuss it further.

*COVID-19 vaccination does not eliminate disease transmission and therefore is not worthwhile.*

Example: Cristina wrote in the online forum that, according to a statement from the CDC, the COVID-19 vaccine did not prevent people from contracting or transmitting the disease. While her statement was true in the sense that no vaccine can completely eliminate disease transmission, a CDC press release on June 7, 2021 stated that COVID-19 vaccines reduce the risk of infection for fully-vaccinated individuals and make the disease milder and shorter in vaccinated individuals who contract COVID-19.

#### Logics of vaccine hesitancy

*Doubts about vaccines stem from the FDA’s accelerated approval process and the lack of available information on the pediatric use of COVID-19 vaccines. Sufficient data have not been collected to demonstrate the safety of the COVID-19 vaccine for young children. Instead of acting hastily and making a wrong decision, it makes more sense to wait for enough information.*

Example: Laura posted in the online forum that she did not trust the safety of the COVID-19 vaccine “yet.” Abigail stated that she was waiting because giving her child the COVID-19 vaccine was an action she could choose to do later, but could not undo. Other mothers agreed that due to the accelerated approval process of the COVID-19 vaccine, this vaccine should not be compared to other childhood vaccines. Instead of outright rejecting vaccines, individuals with some degree of vaccine skepticism may be selective about the vaccines they choose to accept. Sobo et al.^54^ suggest that the multiple, sometimes contradictory positions of selective vaccinators on vaccination are assembled collections that reflect contemporary healing practices to filter information. These healing practices include digitally experienced and collectively constructed “hive” narratives that, according to Goldberg,^43^ are a symptom, not a cause, of public distrust in scientific institutions. While selectively vaccinating parents adopt a curatorial view towards information, engaged healthcare consumers emphasize the need for a non-categorical approach that recognizes the fluid and polyvalent nature of vaccine reasoning.

Our findings suggest that parents may feel more confident about administering the COVID-19 vaccine to their young children if they had access to solid data supporting its benefits. According to existing literature, individuals’ attitudes are influenced by constant changes in public confidence levels regarding vaccines. Larson and Broniatowski^12^ document high volatility in vaccine confidence in the context of COVID-19, suggesting that vaccine confidence levels are influenced by fluctuations in virus surges, as well as additional (mis)information about vaccines. Describing the H1N1 influenza vaccine during the 2009 pandemic, Danielle Ofri termed the passage of feeling characterized by vaccine anxiety, hesitancy, and subsequent rejection of the vaccine as “emotional epidemiology.”^55^

*Personal experiences with the COVID-19 vaccine and each child’s medical history influence vaccination decisions.*

Example: After observing and experiencing breakthrough infections, some mothers in the online focus group expressed dissatisfaction with the effectiveness of the COVID-19 vaccine. Therefore, they were hesitant to vaccinate their children. Other mothers based their decisions on their children’s individual medical histories, such as if the child had asthma and how they had responded to other vaccines in the past.

Parents, in their decision-making, consider each child’s biology, size, susceptibility to specific diseases, environmental hazards, medical and health conditions,^54^ and their own preexisting individual experiences of iatrogenesis.^47^^,^^53^ Gender is an important lens for examining how women from different racial and ethnic backgrounds translate personal experiences of iatrogenesis into vaccine hesitancy for their children.^47^ Additionally, since mothers often play the most significant role in making medical decisions for their young children, these experiences of iatrogenesis are evident in the vaccine schedule that mothers choose to follow for their children.

Brunson and Sobo^56^ found that in California and the state of Washington, rather than merging into marked polarities, parents’ perceptions of childhood vaccination were diverse, dynamic, and multidimensional sets. Likewise, Dubé et al.^15^ assert that parents’ decisions to use vaccination services are multifactorial, heterogeneous, and complex. The processes of risk assessment and management related to vaccines are highly individualized and may be influenced by neoliberal ideologies.^16^

*Mothers hesitant about vaccines do not show complete distrust in scientific institutions.*

Example: In contrast to the public description that vaccine hesitancy stems from ignorance or lack of information, mothers hesitating about vaccines in our focus group engaged in “active reflection,” seeking information from what they consider reliable sources and making responsible and informed decisions. Based on risk perception and value judgments based on their own lives,^57^ they actively sought information from their pediatricians, but did not unconditionally accept it without reservation, instead combining it with “their own research.” That is, they integrated information from the medical institution with information from what they consider other reliable sources. As a result of this process, some mothers emphasized the risk of long-term effects of COVID-19 infection in young children, while others emphasized the risks associated with the COVID-19 vaccine. Our findings highlight the importance of providers partnering with parents and participating in joint decision-making regarding childhood vaccines.

*Parents sometimes encounter contradictory information in medical settings.*

Example: One mother, Elvia, provided a link to an article published by a medical faculty as evidence that COVID-19 vaccines would not have long-term side effects. On the other hand, Patricia, a mother in the online focus group, recounted how her pediatrician told her to wait to get vaccinated against COVID-19 as the vaccine could possibly make her daughters infertile.

Parents face contradictory information in the public sphere, a situation that diminishes credibility and presents the challenge of deciding which sources of information to trust. At the same time, beliefs about vaccines influence how parents interpret vaccination outcomes. For example, Rozbroj et al.^14^ documented reports of permanent infant injuries related to vaccines from parents who believed that some or all vaccines should be rejected. Meanwhile, beliefs about vaccines and autism may not be as extreme as often portrayed in the media. In a study by Anderson-Chavarria and Turner, 32 out of 35 interviewed parents believed that autism is the result of genetic risks triggered by an environmental factor.^58^ Triggers include various environmental pollutants and vaccines; that is, vaccines are considered one of the many possible “triggers” for a child genetically predisposed to autism. While parents may not perceive vaccines as universal, vaccine hesitancy may be indicative of a lack of public trust in science.

Majid and Ahmad^49^ point out how parents rely on information from complementary and alternative medicine providers when they consider the information from allopathic medicine providers to be insufficient. They feel it is easier to have an open and honest discussion with complementary and alternative medicine providers compared to allopathic doctors.

## DISCUSSION

### Competing logics and polyvalent reasoning regarding vaccines

Medical professionals often assume that providing people with the correct information about vaccine safety and the risks of not vaccinating will lead them to make the responsible decision to vaccinate.^11^^,^^59^^,^^60^^,^^61^ However, Roberts and Mitchell^11^ reveal that individuals hesitant to vaccinate take their responsibility for making good health decisions seriously. They seek medical advice, incorporate and evaluate medical information about vaccination, and carefully consider the risks and benefits of vaccinating. Sobo et al.^41^ show that selective parents who did not vaccinate exhibited the type of self-informed participation recommended by the healthcare system. In their study, Rozbroj et al.^14^ state that having children prompted parents to learn more about vaccine options. Similarly, Ward et al.^62^ point out that both vaccine-refusing and vaccine-hesitant families engage in an ongoing search for information on how to make the best medical decisions for their children, leading many to question or distrust Western medical epistemology.

These scholars rely on the reconceptualization by Leach and Fairhead^8^ regarding vaccine hesitancy, which moves away from the conventional “deficit model,” wherein vaccine hesitancy is seen as the result of an individual’s lack of knowledge, trust, or rationality,^43^^,^^51^ and toward an understanding of vaccine hesitancy as “active reflection” (i.e., positive and engaged thinking about how to better care for oneself and others). This reframing shifts the focus from why some people don’t vaccinate to why vaccination doesn’t align with people’s health desires.^8^

In many cases, families who hesitate to vaccinate are particularly proactive when it comes to their children’s health. “Salutogenic parenting,” a term used by Ward et al.,^62^ describes how parents engage in activities that promote health to stimulate natural immunity and protect their children from illnesses. Salutogenic parenting practices include breastfeeding, cooking from scratch, consuming organic and/or homegrown foods, and reducing exposure to preservatives and toxins. These practices overlap with “intensive mothering” - typically associated with white, neoliberal mothers - that is linked to vaccine skepticism.^47^ Parents believe that through these practices, they can reduce or eliminate the need for vaccines. Ward et al.^62^ frame salutogenic parenting as a “care logic” that families internally consider consistent, logically interconnected, and interdependent.

Majid and Ahmad^49^ offer the metaphor of a gear train to understand the overlaps and relationships between various factors that reinforce vaccine hesitancy, refusal, and delay. In their metaphor, each gear represents one of seven factors: “natural” and “organic” life, what Ward et al.^62^ refer to as “salutogenic parenting”; perceptions of other parents; past experiences; sources of information, challenges, and preferences; mandatory vaccine policies; distrust in healthcare system actors; and experiences of interaction with healthcare providers. While these gears work together to drive vaccine hesitations, shared decision-making is a gear that can reverse the mechanism by turning in the opposite direction.

Rozbroj et al.^14^ indicate that having children forces parents to examine their beliefs about vaccines, especially those who hesitate or refuse to vaccinate. Families often do not “accept” or “reject” vaccines altogether but consider vaccines individually, also taking into account children’s biology, size, and susceptibility to diseases.^41^ For some, not vaccinating may be a responsible and informed decision that is “right for them” based on risk perception and value judgments in the context of their lives.^57^ In the case of childhood vaccination, parents classify vaccine-related information as “irregular” and context-dependent. This is especially relevant in the case of the COVID-19 vaccine, as vaccine hesitancy may stem from the FDA’s accelerated approval process and lack of information about the vaccines.

## CONCLUSIONS

While medical and non-specialized press characterize vaccination as a polarizing controversy, qualitative research reveals a multitude of perspectives on the topic. The findings of this study underscore the need to nuance how vaccine acceptance, refusal, and hesitancy are approached, understood, and defined. Vaccine acceptance, refusal, and hesitancy can be considered to operate on a continuum rather than developing into discrete categories. Sorrell and Butler^42^ note that: “Positions once associated with religious or traditional conservative stances have given way to highly disparate views that transcend traditional left/right/religious divisions.”^42^ In the “gray zone” between pro- and anti-vaccine camps, vaccine-hesitant individuals range from “skeptics” to “undecided” or “hesitant.”^9^

In our online focus group, mothers demonstrated fluid and multifaceted reasoning regarding the COVID-19 vaccine. These different lines of reasoning were presented by mothers who allowed their children to receive childhood vaccines, and both lines of reasoning were based on available scientific data (e.g., CDC press releases, research study summaries written for public audiences, and infographics or articles published by medical faculties, etc.) and anecdotal information (e.g., community children contracting a severe case of COVID-19, quickly recovering from a mild case of COVID-19, experiencing breakthrough infection despite being fully vaccinated, or having a reaction to the COVID-19 vaccine). These examples illustrates how information can be interpreted differently depending on parents’ curatorial practices.

Ultimately, families value different sources of information based on their own perception of information reliability and prioritize information that aligns with their personal beliefs.^54^ In the online focus group, Tiffany, a pro-vaccination mother, responded to Roxana, a vaccine-hesitant mother with whom she disagreed, by saying that Roxana should simply say she didn’t want to give the COVID-19 vaccine to her child instead of spreading false narratives. However, my interpretation of the exchange is not that Roxana was trying to spread false narratives, but, rather, that her criteria for what qualifies as reliable information differed significantly from Tiffany’s criteria. Similarly, Elvia responded to Patricia by denouncing the “myth” of infertility as “misinformation used to scare people” and argued that there is no risk. Stephanie refuted that the doctor had pointed out to Patricia the potential risk of infertility. The 5 mothers prioritized information that aligned with their beliefs, experiences, and personal observations of the world around them.

The findings of this study emphasize the different notions of “risk” among mothers of children aged 6 months to 4 years. In the online focus group we observed, some mothers emphasized the unknown long-term effects of COVID-19 infection in young children, while others emphasized the unknown long-term effects of the COVID-19 vaccine (sometimes combined with the known risk of COVID-19; for example, when children had already been infected with COVID-19 and had recovered well from a mild case).

For some individuals, foregoing a recommended vaccine is considered “risky”; for others, accepting the vaccine is seen as “risky.” Viewed through this lens, it’s not that people act carelessly, but rather that individuals with different perspectives are trying to minimize their exposure to risk based on their understanding of what is risky. This approach to vaccine acceptance, refusal, and hesitancy resists the “othering” that is ubiquitous in ongoing debates for and against vaccines. Ultimately, the findings of this study and previous studies indicate that a multitude of factors contribute to vaccine hesitancy among individual parents. Determining, rather than dismissing, the underlying factors in each case should be done with careful consideration.
